# One Health Approach in the Era of Emerging Viruses: The Challenge of Usutu Virus

**DOI:** 10.3390/ijms26178150

**Published:** 2025-08-22

**Authors:** Emanuela Laratta, Domiziana Masci, Anna Caterina Procopio, Alessio Soggiu, Andrea Urbani, Paola Roncada

**Affiliations:** 1 Department of Health Sciences, University Magna Græcia of Catanzaro, Viale Europa, 88100 Catanzaro, Italy; emanuela.laratta@unicz.it (E.L.); annacaterina.procopio@unicz.it (A.C.P.); 2 Department of Basic Biotechnological Sciences, Intensivological and Perioperative Clinics, Università Cattolica del Sacro Cuore, 00168 Rome, Italy; domiziana.masci@unicatt.it; 3 One Health Unit, Department of Biomedical, Surgical and Dental Sciences, University of Milan, Via Pascal 36, 20133 Milan, Italy; alessio.soggiu@unimi.it; 4 Clinical Chemistry, Biochemistry and Clinical Molecular Biology Operations (UOC), Fondazione Policlinico Universitario A. Gemelli IRCCS, 00168 Rome, Italy

**Keywords:** Usutu, zoonosis, omics sciences, viruses, One Health

## Abstract

The One Health concept recognises the close interconnection between human, animal, and environmental health. In recent years, this perspective has intensified scientific focus on zoonoses. Among these, arboviruses—viruses transmitted by arthropod vectors—represent an emerging challenge, particularly in the present period strongly conditioned by climate change. *Usutu virus* (USUV) is a *Flavivirus* maintained via an enzootic bird–mosquito–bird cycle that infects other vertebrates. USUV is currently a significant animal health concern due to its expanding host range and increasing avian mortality events. Although USUV appears to be less dangerous than other emerging arboviruses in humans, the neurological disorders it can cause are alarming and increase the need for a better understanding of the spread and genetic evolution of USUV, as well as for the stronger promotion of vaccine and antiviral development. As with other arboviruses, treatment for USUV is limited to avoiding contact with mosquitoes, which is not always possible. Since vaccines do not yet exist, the use of modern OMICS sciences may provide comprehensive knowledge for developing effective control and prevention measures to avoid future pandemics and contain current epidemics.

## 1. Introduction

Arboviral diseases, zoonoses caused by viruses transmitted by various arthropod vectors, such as mosquitoes, ticks, and horseflies, represent an emerging challenge both due to the expansion of their vector populations, favoured by environmental and climatic factors, and due to the spread of the viruses themselves. The viruses responsible for these diseases belong to different viral families, among which *Flaviviruses* stand out for their clinical relevance and ability to adapt to new ecological and geographical contexts. The recent expansion of these diseases outside endemic areas and the increase in cases of infection have drawn attention to urbanisation, human activity, and other environmental changes that favour the emergence of new outbreaks, demonstrating how human, animal, and environmental health are closely interconnected. For this reason, the World Health Organisation (WHO) has repeatedly emphasised the need to strengthen surveillance and research on emerging viruses for which there are still no specific treatments or established vaccines [1,2,3].

USUV is an emerging mosquito-borne zoonotic virus belonging to the genus *Flavivirus* of the family *Flaviviridae*, which also includes several relevant human pathogens, such as *Dengue virus* (DENV), *Japanese encephalitis virus* (JEV), *yellow fever virus* (YFV), *West Nile virus* (WNV), *Zika virus* (ZIKV), and *tick-borne encephalitis virus* (TBEV) [4,5].

### 1.1. Structure of USUV

Structurally, USUV is a small virus with a diameter of 40–60 nm and a spherical structure consisting of an envelope and an icosahedral capsid containing the viral genome [6,7]. It has an 11 kb positive-sense single-stranded RNA genome with a 5′ capsid but without a 3′ polyadenylated (poly-A) tail [6].

**Figure 1:**
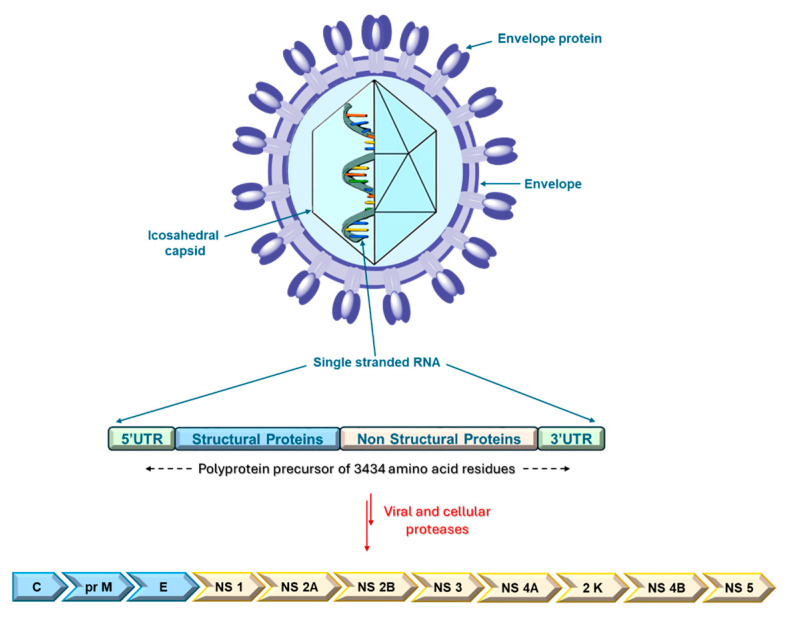
*Usutu* virus virion structure and its genomic organisation.

Like other *flaviviruses*, the genome contains a single open reading frame spanning nucleotides 97–10,401. The USUV open reading frame encodes a 3434 amino acid polyprotein precursor, which is cleaved by viral and cellular proteases to generate three structural and eight non-structural (NS) proteins. The structural proteins (core (C), pre-membrane (prM), and envelope (E)) contribute to viral structural elements, while the NS proteins (NS1, NS2A, NS2B, NS3, NS4A, 2K, NS4B, and NS5) regulate viral replication (Figure 1) [6]. Specifically, NS1 associates with NS4A to enable genomic replication; NS2A coordinates the passage of packaged RNA to replication; NS2B enables the formation of a protease complex with NS3; NS3 is an enzyme with several activities (trypsin-like serine protease, helicase, and RNA triphosphatase, also involved in RNA replication); NS4A associates with NS1 for genomic replication; NS4B is involved in antiviral resistance; and NS5 is an RNA-dependent RNA polymerase (RdRp) [8,9].

### 1.2. Historical Overview of USUV

**Figure 2:**
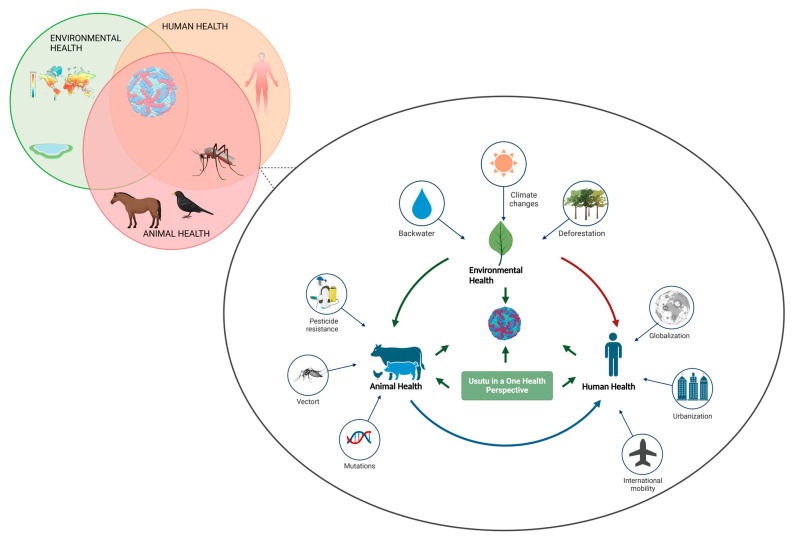
*Usutu* from a One Health perspective.

USUV is closely related to JEV and WNV and was first isolated from a *Culex neavi* mosquito in 1959 in eSwatini (Southern Africa), captured along the Usutu River, from which it takes its name. It was first detected in Italy in 1996, where it caused an epidemic among birds and presumably then spread from Italy to neighbouring countries [10,11,12]. Before 2001, it was considered a minor exotic virus with low pathogenicity, whose circulation was limited to Africa. Subsequently, it spread to different European regions and is now present in most of Europe, causing neuroinvasive disease in birds and, more rarely, in humans. USUV was, in fact, the causative agent in 2001 of the death of many blackbirds in Austria [13]. Phylogenetic studies have investigated the genetic diversity of USUV, categorising its sequences into distinct lineages named according to their geographical origin: Africa 1, 2, and 3, and Europe 1, 2, 3, and 4 [14,15,16,17,18,19]. A recent phylogenetic study, focusing on partial sequences of the NS5 gene (265 bp), revealed a new lineage (Europe 5) composed of viral strains isolated from birds in Germany in 2016 [18]. In recent decades, the virus has become increasingly relevant as a pathogenic agent for humans and animals, primarily due to the expansion of the area in which its vectors are present. This is favoured by climate change, which has enabled mosquitoes to establish themselves in areas where they were previously absent and, thanks to much higher temperatures, extend their period of activity. Moreover, bird migration patterns have altered as a result of climate change, further contributing to the spread of the virus. Thus, the emergence and spread of USUV in Europe exemplify the complex interplay among environmental, animal, and human health factors influencing its transmission dynamics, as outlined within the One Health framework (Figure 2). In line with this pattern, in Italy, each outbreak has caused the death of almost a thousand birds, culminating in the first case of neuroinvasive infection in humans in 2009 [20].

## 2. Transmission

Transmission of USUV has many similarities with that of WNV. The virus is transmitted among birds through the bite of ornithophilic mosquitoes of the *Culex* genus, which also transmit WNV and JEV. In particular, the primary vector in Europe is *Culex pipiens*, while in Africa, the primary vectors are *Culex neavei* and *Culex univittatus* [21,22,23,24,25,26]. USUV has also been isolated in other mosquito species, including *Aedes albopictus* [22], *Aedes caspius*, *Anopheles maculipennis*, *Culex perexiguus*, *Culex perfuscus*, *Coquillettidia aurites*, and *Mansonia africana* [7,25,27]. However, while *Culex pipiens* is considered the primary vector, vector competence has been confirmed under laboratory conditions for *Culex neavei* and *Culex quinquefasciatus* [28,29]. Mammalophilic mosquitoes, such as *Aedes japonicas*, and anthropophilic mosquitoes, such as *Aedes albopictus*, can also be naturally infected, although their vector competence remains relatively weak [30,31,32]. There is currently no data on the ability of USUV to sustain itself in mosquito populations via vertical transmission, unlike WNV, so further studies are needed to determine the role of other mosquito species in USUV transmission.

**Figure 3:**
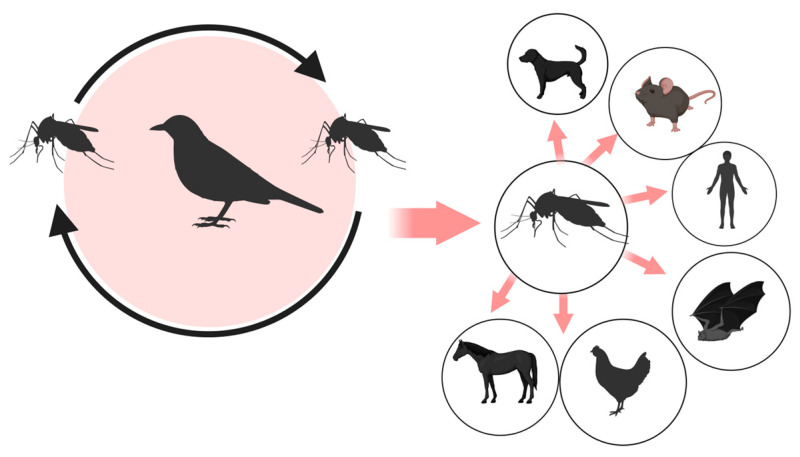
*Usutu virus* transmission. Mosquitoes act as vectors, transmitting the virus primarily between wild birds, which are the main amplifying hosts. Secondary hosts include humans, horses, and other mammals, which can become infected but are considered dead-end hosts. The arrows indicate the possible directions of viral transmission mediated by mosquito bites.

Currently, at least 93 bird species are known to serve as reservoir hosts for USUV. However, some avian species are particularly susceptible to USUV infection with a high mortality rate. This is the case for blackbirds (*Turdus merula*), grey owls (*Strix nebulosi*), and sparrows *(Passer domesticus*) [33,34]. This represents a significant threat to urban areas, as many of the USUV reservoir species are synanthropic, meaning they can adapt to anthropogenic environments. In particular, blackbirds and sparrows are highly adapted to urban life, which could facilitate the circulation of the virus in urban environments; consequently, the impact of USUV concerns avian conservation and public health. The close interaction between synanthropic birds, domestic animals, and humans is not in itself sufficient to cause viral spillover, but it can create favourable conditions that increase the risk of such an event. This highlights the importance of surveillance and control strategies, even though the virus is currently only transmitted through the bite of an infected mosquito (Figure 3).

Although USUV is mainly transmitted through the bite of infected mosquitoes, both extrinsic and intrinsic biological mechanisms influence its ability to persist and spread within vector populations. Trans-stadial transmission, the maintenance of the virus through the different stages of mosquito development (from larva to adult), has not yet been observed in mosquitoes of the Culex genus; adult females usually acquire USUV during blood feeding and do not maintain it during metamorphosis from their aquatic larval stages to their terrestrial adult form. The extrinsic incubation period (EIP), the time required for the virus to replicate and reach the mosquito’s salivary glands, becoming transmissible, depends on temperature and generally varies from 5 to 14 days, with shorter durations observed in warmer environmental conditions. For a long time, vertical (transovarial) transmission, passage of the virus from the adult female to her offspring via eggs, was considered unlikely for USUV, unlike what has been observed for other flaviviruses such as the West Nile virus [35,36,37]. However, recent evidence suggests that vertical transmission may indeed occur, albeit at a low frequency. A study conducted by Schilling et al. detected USUV RNA in male *Culex pipiens* emerging from larvae collected in the wild, providing the first direct indication of the possibility that the virus is transmitted from adult females to their offspring through eggs [38]. However, further studies are needed to confirm its actual epidemiological impact and frequency in different ecological contexts and mosquito species.

USUV can also accidentally infect mammals, including humans. However, mammals are considered accidental or dead-end hosts, as they do not contribute to virus transmission because, unlike some bird species, they develop only low and transient viremia, insufficient to infect mosquitoes. USUV has been isolated from humans, bats, rodents, dogs, wild boar, and several wild ruminants, such as deer, sheep, and roe deer. At the same time, antibodies to the virus have been detected in horses, dogs, squirrels, and even reptiles [39,40,41,42,43].

## 3. Epidemiology

**Figure 4:**
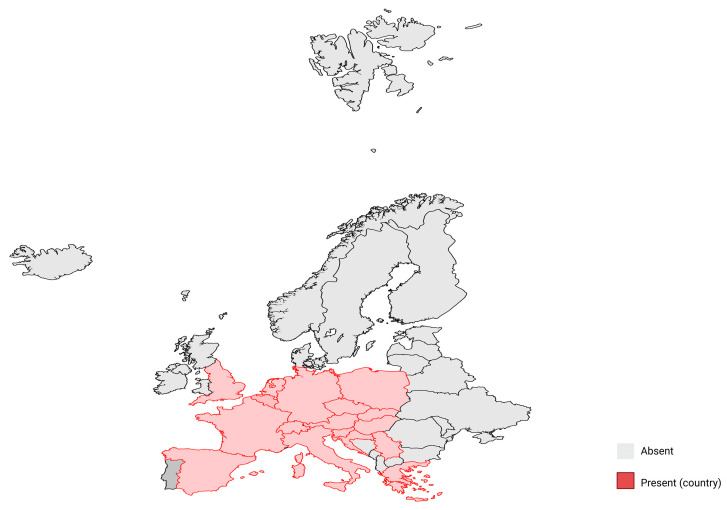
Distribution of USUV in Europe. Countries shaded in red indicate presence of USUV cases (Austria, Belgium, Croatia, the Czech Republic, France, Germany, Greece, Hungary, Italy, Luxembourg, the Netherlands, Poland, Serbia, Slovakia, Slovenia, Spain, Switzerland, and the United Kingdom), while countries in grey indicate absence of reported cases.

Over the past two decades, USUV infection has increased dramatically in several European countries (Figure 4), including Germany, Italy, the Czech Republic, Croatia, Serbia, Spain, Belgium, and France, causing acute mortality in wild birds [44]. A recent study reported the possibility of an intercontinental migration event of USUV from Africa to the Middle East, first described in the late 1990s [45]. To date, USUV has progressively expanded its geographical range across three continents and continues to show the potential for further expansion of its transmission areas [46]. The earliest documented case of human infection with USUV was identified in the Central African Republic during the 1980s [47]. Subsequently, two patients were observed in Africa with fever, rash, and jaundice, and two cases of USUV-positive meningoencephalitis were reported in Italy between 2010 and 2011, leading to the first five cases of USUV-specific IgG-positive sera in healthy blood donors in 2012. The first Italian avian outbreak was reported in 1996 in Tuscany. Ten years later, a high rate of USUV circulation in vectors and birds was reported in Italy. Evidence of USUV’s introduction into Europe since at least 1996 indicated that Italy could be considered the epicentre of the virus’s spread to neighbouring countries [11].

## 4. Pathogenesis and Clinical Manifestations of USUV in Birds and Mammals

The pathogenesis of USUV infection is still poorly understood, although some experimental evidence suggests that it is a neurotropic virus. The clinical course in humans is comparable to that observed for WNV: most infections are asymptomatic or cause mild symptoms. However, symptoms such as fever, rash, headache, and, in rare cases, severe neurological complications such as encephalitis or meningoencephalitis may occur, mainly in immunocompromised individuals [9]. The incubation period is estimated to range from 3 to 12 days following the bite of an infected mosquito. This estimate is primarily extrapolated from WNV infection data due to the limited clinical information available for USUV and the virological similarities between the two viruses. The incubation period is typically followed by a short viraemic phase, during which the virus becomes detectable in biological fluids, such as urine, and aligns with the onset of initial clinical symptoms.

In birds, the predominant vertebrate host species, USUV, can be highly pathogenic. In fact, USUV infection can cause a diverse range of pathological changes that involve numerous organs and systems [6,7,8,9].

The central nervous system is among the most frequently affected, with lesions including non-suppurative encephalitis, necrosis and neuronal degeneration, glial nodules, satellitosis, neuronophagia, and perivascular infiltrates of mononuclear cells. These lesions are commonly observed in the brain and brainstem, while glial arbours, aggregates of microglia around necrotic neurons in the granular layer, may appear in the cerebellum. In some species, vascular damage, such as endothelial cell swelling and vasculitis, is also associated. The viral antigen has been localised within neurons, glial cells, and endothelial cells, suggesting widespread infection of nervous tissue. The liver is another target organ of infection. Macroscopically, hepatomegaly and colour changes are often observed. Histologically, the main features include hepatic necrosis and mononuclear cell infiltration. The viral antigen is mainly concentrated in Kupffer cells, endothelial cells and, more rarely, hepatocytes. The spleen also shows obvious changes: splenomegaly is a frequent finding, associated with zonal necrosis centred on the sheathed arteries and histiocytic hyperplasia. These lesions may appear alone or in conjunction with *Plasmodium* spp. infections, which are common in passerines. The viral antigen has been detected in capsule cells, endothelial cells, and infiltrating mononuclear cells. In the heart, USUV can cause myocardial necrosis and lymphoplasmacytic myocarditis, which can be observed in both passerines and nocturnal birds of prey. In the presence of co-infections with *Plasmodium* spp., hydropericardium has also been described. The virus is mainly localised in cardiomyocytes, endothelial cells, and infiltrating inflammatory elements. In the kidneys, the infection manifests itself as nephromegaly and pallor, accompanied by tubular necrosis and lymphohistiocytic perivascular infiltrates. The viral antigen is detected in tubular epithelial cells, glomerular capillaries, the vascular endothelium, and mononuclear cells distributed in the interstitium. In addition to these main organs, damage to the respiratory and digestive systems has also been documented. In blackbirds, in particular, hyperkeratosis of the periclacal skin has been described, indicative of a skin manifestation associated with USUV [13,33,48].

In humans, USUV infection mainly involves the central nervous system, with clinical manifestations including encephalomyelitis and meningitis. Its effects on the central nervous system can cause severe neurological symptoms, including muscle weakness, ataxia, and convulsions. The virus can also cause injury to the spinal cord and brain, with possible outcomes of lethargic encephalitis and severe neurological dysfunction [48]. In addition, in a study by Simonin et al., more atypical neurological signs were observed, such as peripheral facial palsy and transient sensory disturbances, as reported in a 39-year-old male patient in France who developed acute unilateral facial paralysis, palpebral ptosis, and paraesthesia, without any systemic inflammatory signs or pleocytosis. These findings suggest that USUV-related neurological involvement may encompass a broader and possibly under-recognized clinical spectrum [49].

The tropism of USUV for central nervous system cells is of particular interest in medical research, as it is responsible for significant neurological damage; notably, demyelination of infected neurons has been identified as a distinctive feature of USUV infection [50].

## 5. Treatment and Prevention

Currently, there are no specific antiviral therapies or authorised vaccines against USUV [51]. Therefore, controlling vectors and avoiding bites are the only effective prevention strategies. The development of a vaccine against USUV should be considered a priority. Among the available vaccine platforms, live attenuated vaccines have shown considerable success in preventing *Flavivirus* infections [52]. Two live attenuated vaccines have been approved for human use against other *Flaviviruses*, including the Japanese encephalitis vaccine (JEV SA14-14-2) and the yellow fever vaccine (YFV 17D) [53,54].

A promising strategy for developing live attenuated vaccines involves the creation of highly attenuated and immunogenic chimeric flaviviruses by replacing structural protein genes in the genome of an existing vaccine with corresponding regions of a heterologous flavivirus [55,56]. Using YFV 17D as a backbone, chimeric vaccines against WNV (ChimericVax WN02) and Japanese encephalitis (JE-CV) have been successfully developed [57,58]. The JEV vaccine strain SA14-14-2, licenced in China in 1989, has exceeded 300 million administered doses [59,60]. Due to its excellent safety profile and proven protective efficacy, this strain represents an attractive genetic platform for the design of candidate recombinant flavivirus vaccines. Several chimeric vaccines based on SA14-14-2 have already been described, including candidates against DENV, WNV, ZIKV, and TBE [61,62,63,64,65]. In 2021, Böszörményi et al. explored the development of a subunit vaccine against USUV infection. They showed that the virus envelope protein, expressed in *Escherichia coli*, induces neutralising antibodies against two strains of USUV in rabbits. In addition, neutralising titres were also observed against the closely related WNV. These preliminary results warrant further studies to evaluate the formulation’s protective efficacy. However, significant improvements are needed before it can be considered a valid vaccine candidate [66]. More recently, in 2024, Wang et al. developed a live attenuated chimeric vaccine against USUV (named ChinUSUV) based on SA14-14-2 as a backbone. Its immunogenicity and protective efficacy were evaluated in mouse models, showing that ChinUSUV was highly attenuated in mice and that a single immunisation was sufficient to induce robust protective immune responses against a challenge with chimeric USUV. These results indicate that ChinUSUV may represent a promising vaccine candidate worthy of further development [67].

An additional measure for the prevention and containment of USUV infection involves vector control, particularly of ornithophilic mosquitoes. These strategies include, for example, reducing larval sites by eliminating standing water, using biological larvicides, targeted insecticide treatments, individual protection through repellents and mosquito nets, and the adoption of innovative techniques such as the Sterile insect technique (SIT) and Wolbachia-mediated vector population replacement. Entomological surveillance is also essential for monitoring vector distribution, enabling early identification of outbreaks and timely implementation of appropriate preventive measures [68].

## 6. Diagnosis

**Figure 5:**
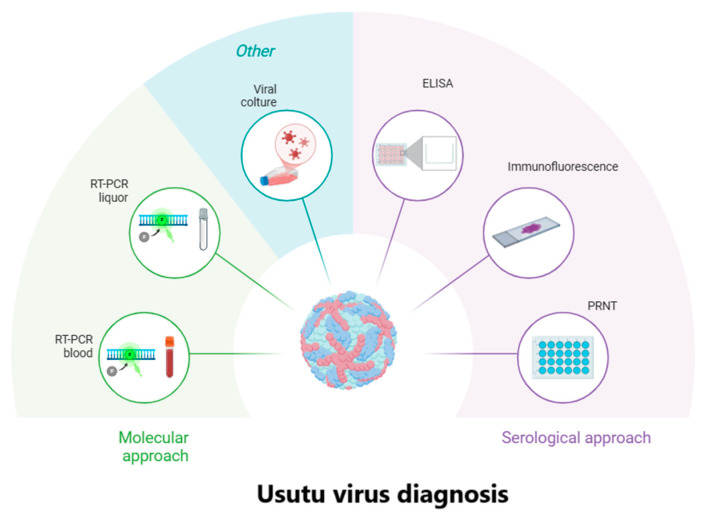
*Usutu virus* diagnosis.

The diagnosis of USUV infection can be conducted using molecular, virological, and serological methods (Figure 5). Molecular diagnosis includes USUV-specific real-time PCR (RT-PCR, wide-range pan-flavivirus PCR and sequencing, or viral isolation in cell culture from blood, urine, cerebrospinal fluid, and any other patient samples, but this is less frequently used due to the complexity and time required when it can be confirmed by PCR or immunofluorescence. Serological diagnosis is based on the detection of the USUV-specific antibody response by immunoassays, such as the ELISA assay, which allows the identification of IgM and IgG antibodies, or the detection of the flavivirus antibody response by cross-reactive serological methods [69]. The serological diagnosis of USUV infection in humans is difficult due to the cross-reactivity of antibodies with other related flaviviruses, such as WNV [70,71,72]. Both serological tests, therefore, require confirmation by neutralisation tests, such as the Plaque Reduction Neutralisation Test (PRNT). This cross-reactivity also underscores the importance of a comprehensive differential diagnosis, aimed at distinguishing USUV infection not only from other neurotropic *Flaviviruses* but also from unrelated viral or bacterial pathogens that may cause similar clinical manifestations.

Similarly, laboratory tests used to identify USUV infection in animals include USUV-specific PCR or wide-range pan-flavivirus PCR and sequencing from blood, tissue, and/or cerebrospinal fluid. Serological diagnosis can be performed by detection of USUV-specific antibody response or flavivirus antibody response using other cross-reactive serological methods (e.g., haemagglutination inhibition, indirect immunofluorescence), followed by a neutralisation test [73].

## 7. Omics Sciences from a One Health Perspective for Vaccine Development

**Figure 6:**
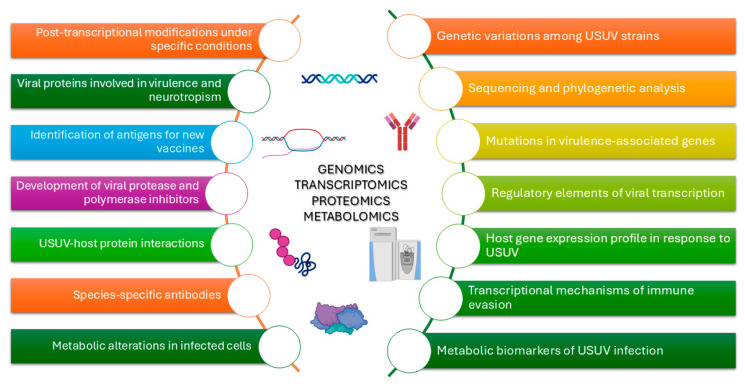
Application of genomics, transcriptomics, proteomics, and metabolomics in USUV research. This image highlights various aspects of analysis, including gene expression, sequence variations, pathogenic mechanisms, immune response, and viral strain typing, emphasising the importance of a multidisciplinary approach to understanding USUV transmission, pathogenesis, and potential control strategies.

Modern omics sciences (genomics, transcriptomics, proteomics, and metabolomics) (Figure 6) are disciplines that play a key role in tackling health emergencies linked to the concept of One Health, particularly for zoonoses caused by emerging viruses such as flaviviruses [74]. Specifically, omics technologies make it possible to identify candidate antigens, study the genetic variability of pathogens to design effective vaccines against different variants, and analyse the immune response by optimising the vaccine formulation. Moreover, genomics and metagenomics are widely used for epidemiological surveillance. In a study conducted by Gaibani et al., the genome of the first USUV isolate from a human patient presenting neurological symptoms was analysed against non-human strains to see if there were any significant differences. Two amino acid substitutions were identified, one in the DIII domain of the E protein and one in the conserved RdRp domain of the NS5 protein, along with unique synonymous substitutions throughout the genome. These changes could explain why this human strain causes encephalitis. However, further studies are needed to confirm how these mutations affect virus behaviour [75]. Proteomics plays a key role in analysing virus–host interactions, making it possible to identify post-translational modifications of viral proteins and understand the molecular mechanisms that regulate their replication [76]. For instance, in a study by Albentosa-González et al., proteomics was essential to identify the phosphorylation of flavivirus NS5 proteins by the cellular kinase Akt [77]. Mass spectrometry made it possible to map the phosphorylated residues precisely and demonstrate that the modification occurs on a conserved Ser in the SGDD catalytic motif of NS5 in ZIKV, USUV, and WNV. However, the effect of phosphorylation varies between viruses: whereas in ZIKV and USUV NS5 mutants, the replacement of Ser with Glu (which mimics phosphorylation) reduced primer extension activity, in the WNV S670E mutant, an increase in this activity was observed. Furthermore, the Ser-to-Glu mutants of all NS5 mutants tested showed almost no de novo RNA polymerisation activity, suggesting a critical role of phosphorylation in the regulation of NS5 function. These results suggest an important role for Akt in replication and encourage further investigations to examine the PI3K/Akt/mTOR pathway as a potential antiviral target, demonstrating how proteomics not only helps to understand the mechanisms of viral replication but can also guide the development of targeted therapeutic strategies against *Flaviviruses*, contributing to the search for effective antivirals in a One Health approach.

## 8. Different Strategies to Mitigate the Impact of USUV Outbreaks

Faced with the growing emergency represented by USUV, the combined use of protease inhibitors (PRIs) and polymerase inhibitors could be an innovative and promising antiviral strategy to be integrated into a One Health approach, which considers and promotes coordinated actions and synergy between human, animal, and environmental health. In this context, the identification and repurposing of existing drugs with established safety profiles might represent an additional and complementary strategy to accelerate the development of effective treatments against USUV, providing a rapid response option in light of the current lack of targeted antiviral therapies.

### 8.1. Development and Use of Protease and Polymerase Inhibitors

PRIs are a class of drugs used in the treatment of certain viral infections, initially developed for the treatment of *human immunodeficiency virus* (HIV) and *hepatitis C virus* (HCV) infections [78]. PRIs act by inhibiting the action of viral proteases, enzymes essential for the maturation of core proteins, because they recognise specific sequences in the long polypeptide strands, performing precise tags that allow protein separation and viral core assembly [79]. By inhibiting this process, protein precursors are not adequately processed, compromising the formation of functional viral particles and halting virus replication [80]. The success of this strategy is exemplified by the development of PRIs such as telaprevir and boceprevir for HCV, highlighting the therapeutic potential of targeting viral proteases. Although these inhibitors have not yet been tested against USUV, their underlying mechanisms have inspired the development of protease inhibitors specifically targeting both USUV and other related *Flaviviruses* (Table 1).

The process of antiviral drug development, which involves many steps such as target identification, high-throughput screening, structure-based drug design, and lead generation and optimisation, has already demonstrated, since the approval of ‘idoxuridine’ in 1963, how the targeted blockade of specific viral functions can result in significant therapeutic advances. Since then, numerous drugs with antiviral potential have been developed for clinical use to treat millions of humans worldwide. However, the ability of viruses to exploit host cells for replication implies considerable difficulties in designing effective and safe drugs, especially considering the variability of viral pathogens [80,81,82,83,84,85]. In addition to protease, polymerase is also a possible target for the treatment of *Flavivirus* infections, as shown in studies for the development of drugs against DENV, as it is the most conserved viral protein in all four serotypes of DENV and is similarly present in USUV [86]. Inhibitors targeting this enzyme have been shown to interfere with the synthesis of the viral genome, thus offering a dual-target approach when combined with PRIs. Experience gained in the development of antivirals for other *Flaviviruses*, such as DENV and WNV, suggests that the simultaneous blockade of protease and polymerase could significantly reduce virus replication and pathogenicity, limiting disease damage and representing a viable alternative to the currently non-existent therapy for USUV [87]. It is important to point out that while for DENV, WNV, and ZIKV, the bibliography contains several studies on PRIs and polymerase inhibitors, for USUV, the literature is still non-existent. The success achieved in the treatment of HCV, an RNA-positive virus belonging, like USUV, to the *Flaviviridae* family, with a genome of approximately 9.6 kB, has shown how the targeting of key components such as the NS3/4A protease, acting in synergy with the NS4A cofactor, and the NS5B polymerase RdRp can lead to effective therapies [88,89,90]. In the HCV genome, ORFs 3′ and 5′ encode, respectively, for the structural proteins (core, envelope glycoproteins [E1 and E2]) that together form the viral particle and for the NS proteins (NS1, NS2, NS3, NS4A, NS4B, NS5A) involved in the viral replication process, whose correct maturation is therefore essential [88,91]. Among the 10 proteins, the HCV protease NS3/4A and the NS5B RdRp are identified as pharmacological targets. The NS3 serine protease is a multifunctional protein with a serine protease and helicase activity that encloses NS4A as its cofactor [89,90]. Consequently, the NS3/4A protein acts together with it to control serine protease activity, recognising the protease as a potential candidate target for ligand design [92,93]. The NS5B protein RdRp plays a key role in viral RNA synthesis and promises to be a highly considered drug target [89,94,95,96,97]. The proven efficacy of inhibitors targeting NS3/4A and NS5B offers a model that could be adapted to other *Flaviviruses*. In fact, several studies have identified small-molecule inhibitors capable of blocking both proteases and the RdRp of related *Flaviviruses*, suggesting that these strategies could be transferred to the treatment of USUV. However, careful structural characterisation of the viral proteins and in-depth testing of their efficacy in vitro and in animal models are required first. For instance, drugs already approved for other viral infections have shown that targeting proteases can result in successful antiviral therapies. A further example is the study by Teramoto et al., in which by high-throughput screening (HTS) of large compound libraries and virtual screening, targeting the proteases of DENV2, WNV, and ZIKV, compounds with novel chemical scaffolds were identified that not only inhibit viral proteases in vitro, but also hinder viral replication in cultured mammalian cells, emphasising how minor structural modifications can improve the binding affinity and stability of antiviral candidates [78,88]. A relevant contribution in this field is offered by the study of del Rosario García-Lozano et al., which described a library of 34 small molecules derived from piperazine, designed through a strategy based on privileged structures involving the incorporation of a 2-substituted piperazine ring, known for its role in activating antiviral activity [98]. The compounds, initially tested by means of commercial HCV NS3/4A protease inhibition assays, showed inhibitory values of over 70% and were subsequently evaluated in vitro for their ability to hinder the replication of viruses belonging to the *Flaviviridae* family. In particular, one of the compounds, characterised by an assembled two-ring structure, showed activity in the low micromolar range and a limited cytotoxic effect; subsequent rounds of optimisation led to the development of two further compounds with three-ring structures, which demonstrated anti-DENV activity superior to the sofosbuvir reference, accompanied by a favourable safety profile. Molecular docking analyses confirmed the interaction of these compounds with protease active sites, reinforcing the hypothesised mechanism of action [98]. The current research on *Flavivirus* PRIs has revealed several compounds with therapeutic potential, some of which act on allosteric pockets of the NS3 protease, preventing activation of the catalytic site. For example, enzyme screening of Food and Drug Administration (FDA)-approved drugs, conducted in a study by Martinez et al., identified molecules such as Zafirlukast, initially developed for asthma, as potential WNV protease inhibitors [87,99]. The search for natural compounds is also proving to be a promising frontier; for instance, Huq et al. pointed out that phenolic compounds extracted from *Theobroma cacao* L., particularly catechin (Table 1), show a high binding affinity for the RdRp of DENV-3, supported by in silico docking, DFT calculations, MD simulations, and MMGBSA analysis [100]. Venkatesan et al. also proposed novel inhibitors for HCV protease and polymerase targets using a virtual screening approach based on e-drugs [95]. In this study, 202 naturally derived compounds including flavonoids, alkaloids, saponins, essential oils, and stilbenes were analysed, supplemented by a large database containing approximately four lakh synthetic compounds with general antiviral activities. The results hypothesised that certain compounds may constitute potential guides for the design of agents capable of inhibiting both NS3/4A and NS5B, thus offering innovative insights into the design of new drugs with enhanced biological activity against HCV, as well as for extending such strategies to other *Flaviviruses*. These results suggest that the natural diversity of molecules may offer innovative solutions for the development of polymerase inhibitors for USUV as well, highlighting how nature itself may offer insights for global health, in line with the principles of the One Health approach. Optimisation of these compounds and their validation as therapeutic agents for USUV could be part of internationally coordinated public health strategies, promoted by the United Nations (UN), which could coordinate research and development efforts to mitigate the impact of epidemics caused by emerging *Flaviviruses* through global surveillance programmes and funding initiatives for antiviral research.

**Table 1:** Representative protease and polymerase inhibitors with potential activity against *Flaviviruses* and *Usutu* virus.

Compound	Target	Virus	Mechanism of Action	Refs.
2-substituted piperazine	NS3/4A protease	HCV, DENV	HCV protease inhibition	[98]
Zafirlukast	NS2B-NS3 protease	WNV	Allosteric inhibition of protease catalytic site	[99]
Catechin from *Theobroma cacao* L.	NS5 RdRp	DENV-3	RdRp inhibition	[100]
CID AE-848/13196185, CID AE-848/36959205, CID 15081408 and CID 173568	NS3/4A protease and NS5B polymerase	HCV	Dual inhibition via virtual screening and docking studies	[96]

### 8.2. Discovery of Repurposed Drugs Against USUV

In light of the emergence of viral threats such as USUV, for which no approved antiviral therapies or vaccines are currently available, alternative and time-efficient drug discovery strategies are urgently needed. Among these, drug repurposing has gained considerable attention as a pragmatic approach to accelerate antiviral development. Unlike de novo drug discovery, which is often time-consuming, costly, and burdened by high attrition rates, repurposing relies on existing compounds with well-established pharmacokinetic profiles, known safety margins, and regulatory approval for other indications. This significantly shortens the timeline required for early-stage drug development and facilitates more rapid progression toward clinical application. Leveraging these advantages, several compounds have recently been investigated for their potential to inhibit USUV replication (Table 2), with encouraging in vitro and in vivo evidence supporting the feasibility of repurposing approaches to address this unmet medical need. A notable example of this strategy was reported by Segura Guerrero et al. in 2018 in an effort to identify promising anti-USUV agents by screening a small set of known flavivirus inhibitors active against WNV and ZIKV, employing validated in vitro antiviral assays and a mouse infection model [101]. Among them, favipiravir (T-705), a broad-spectrum viral RNA polymerase inhibitor, originally developed and approved for the treatment of influenza, was demonstrated to be effective in inhibiting USUV replication when added to the infected cells during the first 6 h after infection. Notably, in an AG129 mouse model infected with USUV (deficient in IFN-α/β and IFN-γ receptors), favipiravir treatment led to reduced viremia and delayed disease progression. These findings highlight the strong potential of favipiravir as a repurposed therapeutic candidate against USUV infection, warranting further clinical investigation. More recently, Wald et al. identified ivermectin, a macrocyclic lactone isolated in 1975 from *Streptomyces* spp. in a Japanese soil sample, as a potent inhibitor of USUV replication [51]. Initially developed and commercialised for veterinary use to treat parasitic infections, ivermectin was subsequently approved by the FDA for human use, notably for the treatment of onchocerciasis (river blindness), strongyloidiasis, and lymphatic filariasis. In this context, its evaluation against USUV represents a clear example of a drug repurposing approach, whereby a compound originally approved for antiparasitic indications is investigated for novel antiviral applications. In vitro studies demonstrated that ivermectin exhibits significant antiviral activity against three cell lines derived from different species, namely as simian (Vero CCL-81), human (A549), and avian (TME R) species, with half-maximal inhibitory concentrations (IC_50_) ranging from 0.55 to 1.94 µM, indicating a strong inhibitory effect on USUV replication. The antiviral mechanism of ivermectin appeared to be multifactorial. For other flaviviruses, including YFV, DENV, and WNV, ivermectin has been shown to exert direct antiviral effects targeting NS3 helicase, an enzyme essential for RNA unwinding and genome replication [102]. Given the extensive structural and functional homology among the non-structural proteins within the *Flavivirus* genus, it is plausible to assume that ivermectin acts through a similar mechanism against USUV. In addition to its direct antiviral activity, ivermectin may also exert host-mediated effects by disrupting the importin α/β-dependent nuclear transport pathway, which several viruses exploit for replication and immune evasion. Inhibition of nuclear transport could therefore represent an alternative or complementary mechanism by which ivermectin attenuates USUV infection. Collectively, these findings support ivermectin as a promising repurposed antiviral candidate for further preclinical and potentially clinical evaluation against USUV infection. Expanding the concept of drug repurposing to include antiviral lead redirection, motivated by the structural similarities among flaviviruses and their conserved viral targets, Chen and colleagues identified two hexahydropyrrolo [1,2-e]imidazol-1-one derivatives, ZDL-115 and ZDL-116, as promising dual inhibitors of both USUV and ZIKV [103]. Although not a repurposed drug in the classical sense, the identification of ZDL-115 and ZDL-116 reflects a repurposing-like strategy in medicinal chemistry, wherein antiviral scaffolds initially designed against DENV and ZIKV are structurally optimised and redirected to target USUV. In vitro antiviral assays demonstrated potent activity in the low micromolar range, with low cytotoxicity and a marked ability to reduce the production of viral progeny. Structure–activity relationship studies highlighted that the incorporation of a 2-deoxyribose moiety into the 3-arene scaffold significantly enhanced aqueous solubility and improved antiviral potency against both ZIKV and USUV. Furthermore, computational molecular docking suggested that ZDL-116 interacts with multiple viral and host targets. These include the ZIKV NS5 methyltransferase (MTase) and NS3 protease, as well as potential binding to the host vitamin D receptor (VDR). Although the mechanistic connection between VDR signalling and the replication cycles of flaviviruses remains to be fully elucidated, this potential host–target interaction opens avenues for exploring immunomodulatory or virus–host interaction-based therapeutic strategies. The discovery and development of such dual-acting anti-flavivirus agents are of considerable significance in the current landscape, where no fully effective antivirals or vaccines exist for many flaviviruses, including USUV. Overall, these findings highlight the importance of drug repurposing and hybrid medicinal chemistry approaches in the prompt identification of promising therapeutic candidates for emerging and neglected viral threats.

## 9. Conclusions

USUV, like the other Flavivirus diseases, represents one of the main examples of One Health challenges, where interactions between humans, animals, and their environments can have major health impacts [104]. The increasing prevalence of USUV, accompanied by its increasing prevalence in humans compared to WNV, especially in Italy, where it has a significant and higher zoonotic potential than the subtypes circulating in other countries, underlines the need for an integrated approach in its management and surveillance [105]. This review underlines the urgency of better characterising the circulating strains through the use of OMICS technologies in order to understand the evolutionary dynamics, transmissibility, and pathogenicity of the virus. Increasing mosquito populations, facilitated by climate change, could further favour the expansion of USUV, increasing the risk of spillover to humans and animals, so early action must be taken. The implementation of adapted, multidimensional surveillance systems capable of simultaneously monitoring USUVs and investment in research into innovative control strategies should be considered to address this emerging threat. OMICS sciences, through the analysis of genomic, proteomic, and transcriptomic data, represent a fundamental tool for the characterisation of the virus and the development of new diagnostic and prophylactic strategies. The application of bioinformatic analysis and big data, tools that are even newer but are showing great potential by providing answers to complex questions and making OMICS even more powerful, can also provide crucial insights into the prediction of transmission patterns and the identification of new therapeutic targets. In this context, the synergy between PRIs and polymerase inhibitors represents a dual-target antiviral strategy of considerable interest for managing emergencies caused by emerging *Flaviviruses* such as USUV. The transfer of knowledge gained in developing drugs for HCV, DENV, WNV, and ZIKV, together with innovation from the use of natural compounds and the optimisation of chemical scaffolds, could enable the development of effective treatments. Achieving concrete results will require a synergetic effort between the scientific community, regulators, and pharmaceutical companies in a context of international cooperation that fully embraces the One Health paradigm, thus strengthening preparedness and response to future global health emergencies.

## Figures and Tables

**Table 2:** Representative repurposed drugs with potential activity against *Flaviviruses* and *Usutu* virus.

Compound	Target	Virus	References
Favipiravir (T-705)	RNA-dependent RNA polymerase	Influenza, USUV	[101]
Ivermectin	NS3 helicase, importin α/β-dependent nuclear transport pathway	USUV, YFV, DENV, WNV	[102]
ZDL-115, ZDL-116	NS5 MTase, NS3 protease, VDR	USUV, ZIKV	[103]

## Abbreviations

The following abbreviations are used in this manuscript:

WHO	World Health Organisation
USUV	*Usutu virus*
DENV	*Dengue virus*
JEV	*Japanese encephalitis virus*
JEV SA14-14-2	Japanese encephalitis vaccine
YFV 17D	Yellow fever vaccine
SIT	Sterile insect technique
YFV	*Yellow fever virus*
WNV	*West Nile virus*
ZIKV	*Zika virus*
TBEV	*Tick-borne encephalitis virus*
Poly-A	Polyadenylated
NS	Non-structural
C	Core
prM	Pre-membrane
E	Envelope
RdRp	RNA-dependent RNA polymerase
RT-PCR	Reverse transcription polymerase chain reaction
PRNT	Plaque Reduction Neutralisation Test
PRI	Protease inhibitor
HIV	*Human immunodeficiency virus*
HCV	*Hepatitis C virus*
FDA	Food and Drug Administration
UN	United Nations
